# GQIcombi application to subdue glioma via differentiation therapy

**DOI:** 10.3389/fonc.2024.1322795

**Published:** 2024-06-26

**Authors:** Varvara Kolesnikova, Alexander Revishchin, Lika Fab, Anna Alekseeva, Anastasia Ryabova, Igor Pronin, Dmitry Y. Usachev, Alexey Kopylov, Galina Pavlova

**Affiliations:** ^1^ Laboratory of Neurogenetics and Genetics Development, Institute of Higher Nervous Activity and Neurophysiology of Russian Academy of Sciences (RAS), Moscow, Russia; ^2^ Laboratory of Neuromorphology, Avtsyn Research Institute of Human Morphology of Federal State Budgetary Scientific Institution “Petrovsky National Research Centre of Surgery”, Moscow, Russia; ^3^ Natural Sciences Center of Prokhorov General Physics Institute Russian Academy of Sciences (RAS), Moscow, Russia; ^4^ Federal State Autonomous Institution «N. N. Burdenko National Medical Research Center of Neurosurgery» of the Ministry of Health of the Russian Federation, Moscow, Russia; ^5^ Chemistry Department, Lomonosov Moscow State University, Moscow, Russia; ^6^ Department of Medical Genetics, Sechenov First Moscow State Medical University, Moscow, Russia

**Keywords:** glioma, G-quadruplex oligonucleotides, antiproliferative activity, small molecule inducers, cancer stem cells, differentiation therapy

## Abstract

Current therapy protocols fail to cure high-grade gliomas and prevent recurrence. Therefore, novel approaches need to be developed. A re-programing of glioma cell fate is an alternative attractive way to stop tumor growth. The two-step protocol applies the antiproliferative GQ bi-(AID-1-T) and small molecule inducers with BDNF to trigger neural differentiation into terminally differentiated cells, and it is very effective on GB cell cultures. This original approach is a successful example of the “differentiation therapy”. To demonstrate a versatility of this approach, in this publication we have extended a palette of cell cultures to gliomas of II, III and IV Grades, and proved an applicability of that version of differential therapy for a variety of tumor cells. We have justified a sequential mode of adding of GQIcombi components to the glioma cells. We have shown a significant retardation of tumor growth after a direct injection of GQIcombi into the tumor in rat brain, model 101/8. Thus, the proposed strategy of influencing on cancer cell growth is applicable to be further translated for therapy use.

## Introduction

Gliomas are diverse and aggressive brain tumors leading to limited survival rates within a few years (1, 2), they are stratified with different Grades The 2021 WHO Central Nervous System Tumor Classification (3) has emphasized molecular characteristics, accentuating the need for their thorough investigations to advance prognosis, treatment and following up. Glioma biomarkers, categorized by location and function (4), provides potential insights, yet specific markers remain controversial ones.

Conventional therapies like surgery, radiotherapy, and chemotherapy scarcely prevent tumor recurrence due to glioma’s invasive nature (5). Various strategies using advances in immune therapy, gene and cell therapies (6–9), have been applied, though a cure remains elusive.

Resistant cancer stem cells drive tumor recurrence due to complex influences like microenvironment, signaling, and surface markers (10, 11). Attacking these cells is pursued through targeting agents (12), requiring in-depth approaches to rule out survival of cancer cell.

Cell undergoes programmed steps in cell cycle, guided by specific regulators, which results in either proliferation or differentiation and they are mutually exclusive (13). By manipulating with regulators, it is possible to reprogram the cell. For example, ectopic expression of cyclins and CDKs enhances reprogramming, while depletion has the opposite effect (13).

Tumorigenesis disorganizes a normal proliferation process and changes the proliferative index (14, 15). A reprogramming cell from proliferation to differentiation could correct aberrant cell division.

Cancer cell could be reprogrammed with entities like small molecules, microRNAs, transcription factors, exosomes, others (16). Therapy, which direct cancer cells toward non-cancer states, like ZNF117-mediated glioma stem cell differentiation (17), provides hope but requires further studies for optimization.

Other approach of successful reprogramming is a halting of proliferation. Well-known blockers, antibody-based drugs, like ADCs, could not be used because of a toxicity for the cells (18). Von Knebel Doeberitz et al. proposed a non-toxic approach using epigenetic changes to drive glial lineage-maturation through pharmacologic inhibition of repressive enzymes (19). Aptamers, synthetic oligonucleotides, can be antiproliferative agents with benefits including size, flexibility, rapid production, stability, and low immunogenicity (20). Cytostatic G-quadruplex (GQ) oligonucleotides have been effective on glioma cell cultures (21), showing promise in this context (22, 23).

Translation from *in vitro* to *in vivo* has to consider a dosing efficiency and injection methods. Animal models with precise injection techniques are crucial for a developing of optimal anti-tumor approaches. Given that many treatments target specific cancer cell types, our strategy has demonstrated the efficacy throughout various glioma Grades (II to IV). The GQIcombi treatment causes the significant reduction of the tumor size for glioma rat models, viewing with MRI.

In our previous study, we had demonstrated a retardation of proliferation of glioblastoma cell cultures, Grade IV, after GQIcombi treatment: cytostatic G-quadruplex bi-(AID-1-T) oligonucleotide (21), SB431542 (SB), purmorphamine (PRM), LDN-193189 (LDN), and Brain-Derived Neurotrophic Factor (BDNF) (24). SB431542 acts as a competitive inhibitor of the ATP-binding sites of activin receptor-like kinases 4 (ALK4), ALK5, and ALK7, which are type 1 receptors in the TGF-β pathway, thereby specifically blocking Smad2/3-mediated signal transduction (25, 26). SB431542 induces the differentiation of human embryonic stem cells (ESCs) and induced pluripotent stem cells (iPSCs) (26, 27). LDN-193189, an inhibitor of Bone Morphogenetic Protein (BMP) signaling, is used alongside SB431542 for neural induction. Elevated BMP4 expression during oncogenesis influences the proliferation of glioma stem cell (28). Purmorphamine, utilized as an analog of the Sonic Hedgehog protein, has proven effective in generating motor neurons from human induced pluripotent stem cells (29). BDNF stimulates the development and differentiation of new neurons (30) and acts as a neural inducer during the final stage of neural cell maturation (31). The abovementioned molecules have been shown to stimulate neural differentiation in the protocols of turning of iPSCs and adipose tissue-derived mesenchymal stem cells into mature dopaminergic neurons (32, 33). In this research, we have proved the efficacy of the GQIcombi treatment regimen to inhibit a proliferation throughout diverse glioma Grades.

## Materials and methods

### Primary cultures of glioma cells

The studies involving human participants were reviewed and approved by The Local Ethics Committee of N. N. Burdenko National Medical Research Center of Neurosurgery. The patients/participants provided their written informed consent to participate in this study. Cell cultures G01, Sus (Sus\fP2), Bl (G23), Rozh (G22), 40, Sh\fP3 (N1) (IV Grade), 22, 42, G11 (III Grade), G13 (II Grade) were obtained from human glioma tissues after tumor resection. Upon tumor resection cells were treated as it is previously described (24).

### Cell cultivation with aptamers and small molecules

In *in vitro* experiments following concentrations of GQIcombi were used: bi-(AID-1-T) (37,5 μM), LDN-193189 (1 μM, Miltenyi Biotec, Germany), SB41542 (10 μM, Miltenyi Biotec, Germany), PRM (2 μM, Miltenyi Biotec, Germany), BDNF (20 ng/mL, Miltenyi Biotec, Germany). Before adding them to the cell cultures, aptamers were pre-formed at 95°С and cooled overnight at 4°С.

Cultures received treatment as follows: On day 1, bi-(AID-1-T) was introduced to the medium. On day 3, SB431542 and LDN-193189 were added. On day 5, Purmorphamine was introduced alongside bi-(AID-1-T)+SB431542+LDN-193189. On day 7, BDNF was added to bi-(AID-1-T) +SB431542+LDN-193189+Purmorphamine or bi-(AID-1-T) only flasks. For simultaneous treatment, bi-(AID-1-T) was added on day 1 and SB431542+LDN-193189+Purmorphamine+BDNF on day 3. Alternatively, all factors were added on day 1. The scheme was modified from the protocol of conversion of human astroglial cells into functional neurons (34).

### MTT assay

Proliferation activity changes in glioma cells post bi-(AID-1-T) and small molecule exposure were assessed using MTT assay. Glioma cells (800–1500 cells per well) were seeded in 96-well plates (three replicates per concentration) with DMEM/F12 medium. Incubation occurred at 37°C with 5% CO_2_ for 10 days. On day 10, cells were washed, and 100 μl culture medium plus 10 μl MTS reagent (Promega, USA) were added per well. Incubation followed for 2 hours at 37°C with 5% CO_2_. Positive controls lacked aptamers; cell medium served as a blank. Optical density was measured at λ = 495 nm using a CLARIOstar Plus plate analyzer (BMG LABTECH, Germany).

### Cell survival

Experiments utilized xCELLigence Real-Time Cell Analysis (RTCA) S16 Instrument (ACEA Biosciences, USA) with 16-well plates (E-plate 16 PET, ACEA Biosciences, USA). Each concentration was tested in duplicate with a seeding density of 5–6×10^4^ cells/well in DMEM/F12 medium. Impedance measurements were conducted following the protocol outlined in ‘Cell cultivation with aptamers and small molecules’. Experiments ran for 240 hours, recording measurements every 15 minutes within a CO_2_ incubator, connected to a laptop. Cell index, reflecting cell adhesion, viability, morphology, and proliferation, was analyzed. RTCA Software Lite was used to undergo data registration and analysis.

### Immunocytochemistry

Glioma cells of II, III and IV Grades at a concentration of 2×10^4^ cells/slip were cultivated on cover slips for culture plates SPL Lifesciences, USA) in 24-well plates in DMEM/F12 medium with 1% glutamine and 10% FBS. After exposure to GQIcombi as described above cells were washed once in PBS (pH 7.3) and then fixed in 4% paraformaldehyde solution for 20 min at 4°C. Cells were then washed twice more in PBS (pH 7.3).

Staining was performed using the following primary antibodies: mouse monoclonal anti-CD133 antibody (1:20, Miltenyi Biotec, Germany), rabbit polyclonal anti-Oct4 antibody (1:100, Abcam, UK), rabbit polyclonal anti-Nestin antibody (1:50, Chemicon, USA), goat polyclonal anti-Sox2 antibody (1:20, Santa Cruz, USA), rat monoclonal anti-CD44 antibody (1:100, Invitrogen, USA), goat polyclonal anti-Notch1 antibody (1:50, Santa Cruz Biotechnology, USA), rabbit polyclonal anti-L1CAM antibody (1:100, Thermo Fisher Scientific, USA), rabbit polyclonal anti-ki67 antibody (1:100, Abcam, UK), rabbit polyclonal anti-bIII-tubulin antibody (1:100, Abcam, UK), and mouse monoclonal anti-NeuN antibody (1:50, Chemicon, USA). The primary antibodies were dissolved in PBS with 0.3% Triton X100 (Sigma-Aldrich, USA) as a detergent and 2% donkey serum (Jackson Immunoresearch, UK) and incubated for 1 h at room temperature.

After triple washing with PBS (pH 7.3) for 5 minutes, cells were exposed to the following antibodies: donkey anti-rabbit Alexa Fluor 488 (1:100), donkey anti-goat Alexa Fluor 488 (1:100), donkey anti-rat Cy2 (1:100), and donkey anti-mouse Alexa Fluor 594 (1:100), all from Jackson Immunoresearch, USA. Subsequently, cells were PBS-washed and stained with bisbenzimide (1:500, Hoechst, Sigma-Aldrich, USA) for 5 minutes at room temperature. After additional PBS washing, cells were fixed for 8 hours at +4°C in Mowiol 4–88-based confinement medium Sigma-Aldrich, Germany) containing 1% DABCO antioxidant, followed by analysis using fluorescent microscopy.

### Apoptosis analysis

Glioma cells were cultured in 24-well plates with DMEM/F12 medium containing 1% glutamine and 10% FBS. On days 8, 15, 21, and 30, cells were washed with PBS (pH 7.3) and fixed in 4% paraformaldehyde at 4°C for 20 minutes. After two PBS washes, cells were incubated with primary rabbit anti-Caspase 3 antibodies (1:100, Sigma-Aldrich, USA) in PBS containing 0.3% Triton X100 and 2% donkey serum for 1 hour at room temperature. Following three 5-minute PBS washes, cells were exposed to donkey anti-rabbit Alexa Fluor 594 antibodies (1:50, Jackson Immunoresearch, USA) for 1 hour. Subsequently, cells were PBS-washed and stained with bisbenzimide (1:500, Hoechst, Sigma-Aldrich, USA) for 5 minutes at room temperature. Cells were then fixed with Mowiol 4–88-based confinement medium (Sigma-Aldrich, Germany) containing 1% DABCO antioxidant at +4°C for 8 hours, and analyzed through fluorescent microscopy.

### Confocal microscopy

Confocal microscopy was performed using a complex of laser scanning confocal microscope Carl Zeiss LSM-710 (ZEISS, Germany) series with a short-pulse femtosecond infrared laser with a tunable range (800 - 1500 nm) for multiphoton excitation of fluorescence.

### Transcriptome analysis

G01 glioblastoma cells underwent GQIcombi exposure as described above. For transcriptomic analysis, cells were treated on the 8^th^ day with Trisol reagent (Thermo Fisher Scientific, USA) following a protocol. Total RNA quality and quantity were assessed using BioAnalyzer and RNA 6000 NanoKit (Agilent, Germany) (**Supplementary Figure 1**). Subsequently, polyA fraction isolation from total RNA employed Dynabeads^®^ mRNA Purification Kit (Ambion, Thermo Fisher Scientific, USA) oligoT magnetic beads per the kit’s protocol. PolyA RNA libraries were then generated using NEBNext^®^ RNA UltraII (NEB, UK) kit, quantified using Qubit dsDNA HS Assay Kit (Thermo Fisher Scientific, USA) on a Qbit 2.0 instrument (**Supplementary Table 1**), and fragment length distribution analyzed with Agilent High Sensitivity DNA Kit (Agilent, Germany) (**Supplementary Figure 2**). Sequencing was executed on a HiSeq1500 instrument (Illumina, USA), producing a minimum of 10 million 50-nucleotide short reads per sample (**Supplementary Table 2**).

Next, differentially expressed genes were calculated using the following algorithm:

1. Mapping of reads to the genome: Mapping was performed by the STAR program version 2.7.9a with an additional parameter -outFilterMismatchNmax 3, GRCh38 genome, Ensembl annotation;
2. Differential expression analysis: DESeq2.0.

### RT-qPCR

Expression of the following markers was measured in glioma cell cultures by RT-qPCR: *CD24, EPHA3, BMP4, WNT5, TNC, CD133, CD44, Nestin, L1CAM, Sox2*, *Notch2*, *bIII-tubulin* and *NeuN*. The TRIzolTM Reagent (Sigma-Aldrich, USA) was used for RNA isolation. DNA strand synthesis was performed using MMLV RT kit (Evrogen, Russia). G01 cells were treated with GQIcombi. The initial G01 culture was used as a control. The assay was performed under the following conditions: preliminary warming for 5 min at 90°C, denaturation for 20 sec at 94°C, primer annealing 12 sec at 60°C and elongation for 15 sec at 72°C. The number of cycles was set to 41. Each sample was carried out in triplicate. The primers used in the assay are presented in **Table 1**. The house-keeping genes used in the research were GAPDH and HPRT. RT-qPCR was carried out using a LightCycler 96 amplifier (Roche, Switzerland).

**Table 1:** Panel of primers for RT-qPCR.

*CD24*	GAAGAGAGAGTGAGACCACGAAG CAG TGAAACAACAACTGGAACTTCA
*EPHA3*	AGACACGGTACCCATGGACT GCATTGCAGGAACACTTGCC
*BMP4*	AGTCATTCCAGCCCACATCG CTGGTCACCTTTGGCCATGA
*WNT5A*	GCTGCAGTTCCACCTTCGAT ACTGGCAGGACTTTCTCAAGG
*TNC*	TCTTTGGCTGGGTTGCTTGA TGGCATTGAGCTGACCTACG
*CD133*	TGGATGCAGAACTTGACAACGT ATACCTGCTACGACAGTCGTGGT
*CD44*	CCCCAGCAACCCTACTGATG GCCTCTTGGTTGCTGTCTCA
*Nestin*	TTGCCTGCTACCCTTGAGAC GGGCTCTGATCTCTGCATCTAC
*L1CAM*	CATGTGATGGAGCCACCTGT CCCAGCTCTTCCTTGGGTTT
*Sox2*	GCTCGCAGACCTACATGAAC GGGAGGAAGAGGTAACCACA
*Notch2*	TGTGACAGCCTGTATGTGCC ACCCCTCCATTCTGACACCT
*bIII-tubulin*	GGAGATCGTGCACATCCAGG GATGTCCAAAGGCCCCTGAG
NeuN	TACG CAGCCTACAG ATACGCTCA ATGGTTCCAATGCTGTAGGTCG
*HPRT*	TGAGGATTTGGAAAGGGTGT GAGCACACAGAGGGCTACAA
*GAPDH*	AGATCCCTCCAAAATCAAGTGG GGCAGAGATGATGACCCTTTT

### Animals

Adult male Wistar rats (200–250 gg., n=12) were used as experimental subjects. Animals were obtained from the “Stolbovaya” nursery of the FMBA Scientific Center for Biomedical Technologies (Stolbovaya Settlement, Chekhov District, Moscow Region, Russian Federation).

All rats were kept under a 12/12 h day–night cycle. Animals were housed in standard plastic cages in groups of 4–5 animals per cage. Food and tap water were available ad libitum.

Experiments were performed according to the European Union Directive 2010/63/EU on the protection of animals used for scientific purposes. Animal care and use were in accordance with the institutional policies and guidelines. The study was approved by the Ethical Committee of the IHNA (protocol 007 from 12.08.23). All efforts were made to minimize the number of animals used in experiments and their suffering from experimental procedures.

### Evaluation of the antitumor activity of the bi-(AID-1-T) aptamer in combination with small molecules against glioblastoma in 101/8 tumor rat model

The tumor model was kindly provided by A.S. Khalansky from Research Institute of Human Morphology (Moscow, Russia). Glioma 101/8 was initially produced by local injection of DMBA (7,12-Dimethylbenz[a]anthracene) and maintained by repeated intracerebral implantation. This model shows high similarity to human glioblastoma in its histological characteristics (35). The transplantation of 101/8 glioma was performed using tumor tissue. For tumor reconstitution, cryostored ampoules were rapidly thawed at 37°C and centrifuged to extract cells from the cryoprotectant. Tumor tissue (appr. 0.8–1.0 x10^6 cells) was then implanted into rat brains (n=4) using an insulin syringe and a 0.8 mm needle. Rats were anesthetized with zoletyl-xylazine ((tiletamine hydrochloride- 5 mg/kg, zolazepam hydrochloride- 5 mg/kg (“Zoletil 100”, Virbac, France), xylazine hydrochloride 1.08 mg/kg (“Xyla” Interchemie, Netherlands), i.p.), and a skin incision was made on the skull. A 1.8 mm diameter hole, 2 mm caudal of the bregma line, was created with a dental burr. A suspension of 1 million cells was implanted 4 mm deep from the outer skull surface into the third ventricle bottom using an insulin syringe. The wound was sutured, and animals were observed. These animals were not used for experiments but as a tumor source. After 14–21 days, rats displayed tumor-related symptoms (weight loss, tremors, convulsions). The animals were sacrificed by inhalation overdose of 5% isoflurane (Laboratories Karizoo. Spain), the brain and the tumor were removed under aseptic conditions.

### Implantation of 101/8 glioma cells in the brain of a rat

The donor animal’s tumor was crushed and tumor tissue was injected into experimental animals (n=6) using a trocar at identical coordinates (appr. 0.8–1.0 x10^6 cells). Surgical suturing followed. Animals were housed conventionally until the experiment. Post-tumor implantation, animals were randomized into two groups (n=3 each): control (no therapy) and “experimental” (therapy with aptamer and factors, per **Table 2**). Antitumor efficacy was assessed via MRI (Bruker Biospec 7 T, Germany) on days 7 and 14 pre- and post- therapy. Images of the tumor obtained using T1-mode MRI with contrast agent Gadovist (Bayer AG, Germany) injected in tail vein in dose 0.5mmol/kg directly before the examination. The tumor volume was measured by calculating the tumor area on serial tomograms obtained in 0.8 mm increments. Glioma 101/8 - implanted animals were euthanized on day 14 by inhalation overdose of 5% isoflurane. Brains were frozen, sectioned (10 μm) on a cryostat, and fixed with 4% paraformaldehyde in PBS.

**Table 2:** Scheme of the experiment of introduction GQIcombi (bi-(AID-1-T) + SB431542, LDN-193189, purmorphamine, and BDNF) into rat glioma 101/8.

Day of therapy after tumor implantation	Factor and dose
8	bi-(AID-1-T)– 37,5 µМ
9	bi-(AID-1-T) – 37,5 µМ + SB431542 (20 µМ) + LDN-193189 (2 µМ)
10	SB431542 (20 µМ) + LDN-193189 (2 µМ) + purmorphamine (4 µМ)
11	purmorphamine (4 µМ) + BDNF (40 ng/ml)

### Assessment of the accumulation of the bi-(AID-1-T) in glioblastoma 101/8 on 14^th^ day of tumor growth

Male Wistar rats (n=2) were implanted with glioblastoma 101/8, following prior protocol.

On the 14^th^ day of tumor growth, FAM-labeled bi-(AID-1-T) was tail vein-injected at 3.6 µmol/kg. After 15 minutes, animals were euthanized using isoflurane overdose. Brains were frozen on Peltier elements, cryostat-sectioned (10 µm), and fixed in 4% paraformaldehyde. Sections were bis-benzimide-stained. Accumulation in tumor tissues was analyzed using Carl Zeiss LSM-710 laser scanning confocal microscope with multiphoton excitation (wavelengths: 420 nm for FAM-label, 365 nm for bis-benzimide) analyzed bi-(AID-1-T).

### Statistical analyses

MTT assay analysis employed MARS Data Analysis Software, and target gene expression levels were measured using LightCycler^®^ 96 System Software. Statistical analysis used GraphPad Prism 9, with data presented as means ± SD. MTT significance was evaluated through one-way ANOVA followed by Bonferroni’s multiple comparisons test. RT-qPCR results were assessed using multiple unpaired t tests to compare experimental and control group means. Figures indicate significance as follows: *= p<0.05, ** = p<0.01, *** = p<0.001, **** = p<0.0001.

## Results

As gliomas of II, III and IV Grades have diverse features when cultured it is expected that they will react to GQIcombi exposure differently. So, IV Grade gliomas are characterized to actively proliferate comparing to the II Grade gliomas (36).

To prove the concept of use an antiproliferative G-quadruplex together with small molecules GQIcombi by its ability to decrease cell proliferation it was tested on glioma cells of different Grades. Following glioma cell cultures, derived from patients after tumor resection, were used (**Table 3**).

**Table 3:** List of patient glioma cell cultures with brief description.

Cell Culture	Glioma Grade	IDH1 Status	Patient gender	Tumor location	Citations
G01	IV	Wild type	M	Left frontal lobe	(21, 24, 37, 38)
Sus (Sus\fP2)		Wild type	F	Left temporal lobe	(21, 24, 38)
Bl (G23)		Wild type	M	Left frontal lobe	(21, 37)
40		Wild type	M	Left temporal lobe	(24)
Rozh (G22)		Wild type	F	Left frontal lobe, corpus callosum, interventricular septum	(21, 24)
Sh\fP3 (N1)		Wild type	M	Right motor-premotor area	(21)
G11	III	Wild type	F	Right frontal, temporal, insular lobe	(21, 37)
22		Wild type	M	Right temporal, insular lobe	
42		Mutation	M	Right frontal, temporal, occipital lobe	
G13	II	Wild type	M	Right frontal, insular lobe	

### GQIcombi inhibits crucial signaling pathways and genes required for cancer cell maintenance

Transcriptome analysis assessed changes in gene expression, associated with proliferation, migration, and gene expression linked to cancer cell maintenance. Glioblastoma culture G01 and GQIcombi were used, yielding differentially expressed genes with padj<0.01 (GEO accession GSE250617). Differential expression results are categorized in heat maps by cellular function.

**Figure 1:**
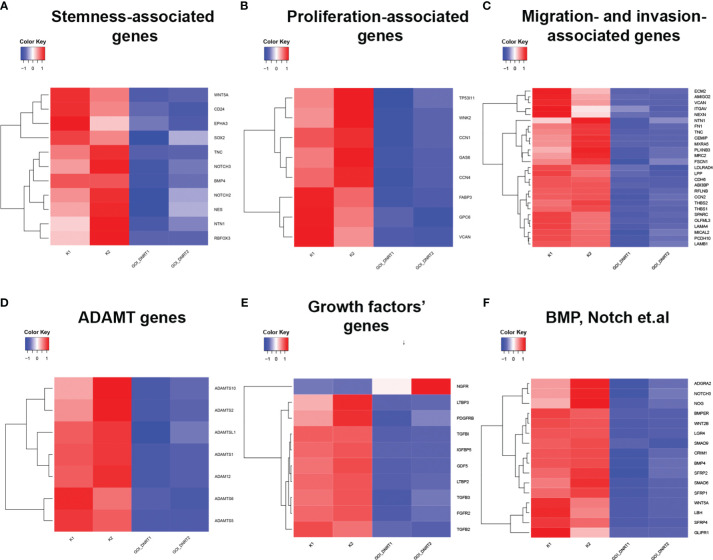
Transcriptomic analysis of target genes upon GQIcombi (bi-(AID-1-T) + SB431542, LDN-193189, purmorphamine, and BDNF) exposure. Transcriptomic analysis of G01 glioma cell culture after exposure to GQIcombi. **(A)** Alteration of stem cells associated genes. **(B)** Alteration of genes associated with proliferation. **(C)** Alteration of genes associated with migration and invasion. **(D)** Alteration of ADAMT genes. **(E)** Alteration of growth factors genes. **(F)** Alteration of genes responsible for stem cell maintaining.

Transcriptomic findings (**Figures 1A–F**) indicate reduced stemness, proliferation, and migration gene expression with GQIcombi, suggesting decreased tumor aggressiveness. Expression decline in stemness signaling factors (Notch, WNT/b-catenin), and cancer stem cell proliferation promoters (BMP, Gli), reflects effective small molecule inhibition of these pathways. An RT-qPCR panel was designed based on stemness-associated genes.

### GQIcombi inhibits cell proliferation and decreases stemness-related gene expression in II-IV glioma cell cultures

**Figure 2:**
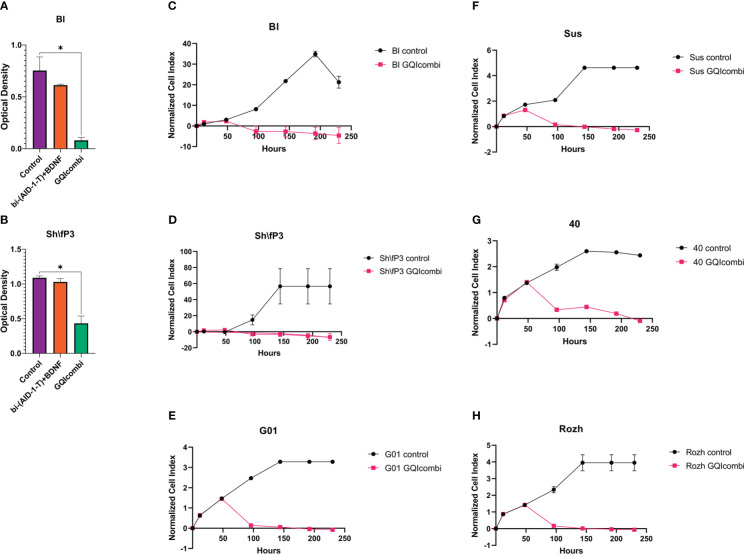
GQIcombi (bi-(AID-1-T) + SB431542, LDN-193189, purmorphamine, and BDNF) influences proliferation features of IV Grade glioma cell cultures Bl, Sh\fP3, G01, Sus, 40 and Rozh. **(A, B)** MTT assay for Bl and Sh\fP3 cell cultures in 10 days after the exposure to GQIcombi. Statistically significant differences between the control and the treatment groups are indicated by asterisks (One-Way ANOVA, Bonferroni’s multiple comparisons test, *= p<0.05). **(C–H)** Plot of normalized cell index versus time of Bl, Sh\fP3, G01, Sus, 40 and Rozh cell cultures in 10 days after the exposure to GQIcombi. Data are represented as mean ± SD; n = 2 for each group.

**Figure 3:**
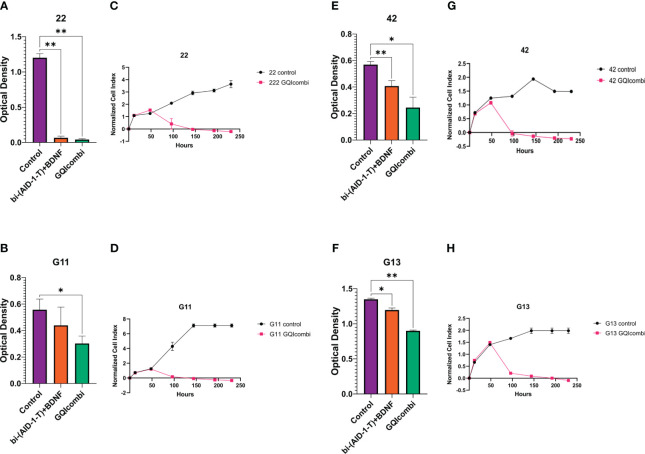
GQIcombi (bi-(AID-1-T) + SB431542, LDN-193189, purmorphamine, and BDNF) influences proliferation features of III Grade glioma cell cultures 22, G11, 42 and II Grade glioma cell culture G13. **(A, B, E, F)** MTT assay for 22, G11, 42 and G13 cell cultures in 10 days after the exposure to GQIcombi. Statistically significant differences between the control and the treatment groups are indicated by asterisks (One-Way ANOVA, Bonferroni’s multiple comparisons test, *= p<0.05, ** = p<0.01). **(C, D, G, H)** Plot of normalized cell index versus time of 22, g11, 42 and g13 cell cultures in 10 days after the exposure to GQIcombi. Data are represented as mean ± SD; n = 2 for each group.

Characterization of glioma cell cultures of Grades IV, III, and II employed MTT assay, XCelligence Real-Time Cell Analyzer, ICH, and RT-qPCR. MTT assay was conducted on day 10, excluding G01, Sus, 40, Rozh (previously reported (24),). XCelligence RTCA S16 involved six analysis time points, including factor additions at 24-hour intervals, with a key impact observed upon bi-(AID-1-T) addition (**Figures 2C–H**, **3C, D, G, H**).

RT-qPCR examined stem cell and neural maturation gene expression to confirm changes induced by small molecules promoting cancer stem cell neural differentiation. Immunocytochemical staining after 10 days incubation assessed target protein alterations, including NeuN and bIII-tubulin for neural maturation and ki67 for proliferation.

*IV Grade*

Bl

MTT: significant inhibition of cell proliferation activity; cell proliferation decreased on 25% after exposure to bi-(AID-1-T)+BDNF and on 90% after exposure to bi-(AID-1-T)+SB, LDN, PRM+BDNF (**Figure 2A**).

**Figure 4:**
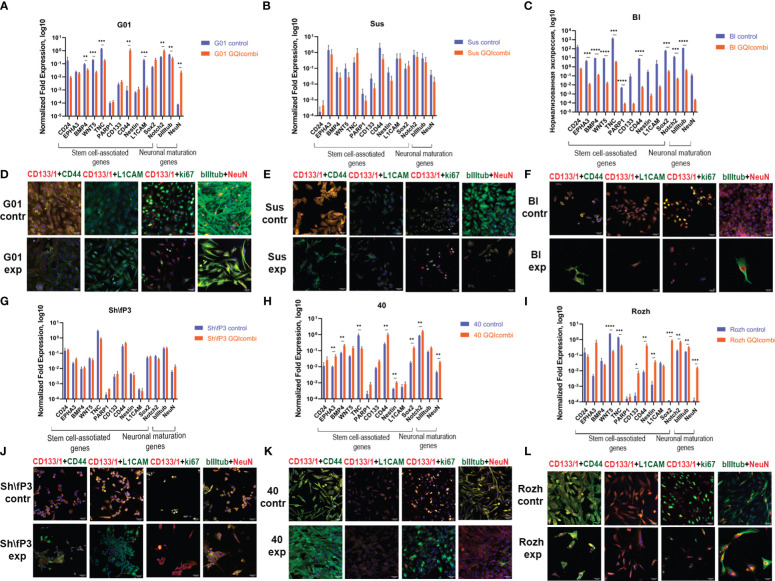
GQIcombi (bi-(AID-1-T) + SB431542, LDN-193189, purmorphamine, and BDNF) influences stemness features of IV Grade glioma cell cultures G01, Sus, Bl, Sh\fP3, 40 and Rozh. **(A–C, J–L)** The expression of neural stem cells’ genes in G01, Sus, Bl, Sh\fP3, 40 and Rozh cell cultures cultures in 10 days after the exposure to GQIcombi. Data are represented as mean ± SD; n = 3 for each group. Statistically significant differences between the control and the treatment groups are indicated by asterisks (multiple unpaired t test, *= p<0.05, ** = p<0.01, *** = p<0.001, **** = p<0.0001). **(D–F, G–I)** Micrographs of immunocytochemical staining of G01, Sus, Bl, Sh\fP3, 40 and Rozh cell cultures with anti-CD133, anti-CD44, anti-L1CAM, anti-ki67, anti-bIII tubulin and anti-NeuN antibodies before (contr) and after (exp) exposure to GQIcombi. Scale bar is 50 μm.

RT-qPCR: Bl has shown to decrease expression of all tested genes (**Figure 4C**).

ICH: CD133 (↓), ki67 (↓) (**Figure 4F**), Notch1 (↓) and Oct4 (↓) (**Supplementary Figure 3**) decreased expression levels were observed.

Sh\fP3

MTT: significant inhibition of cell proliferation activity; cell proliferation decreased on 9,1% after exposure to bi-(AID-1-T)+BDNF and on 63,6% after exposure to bi-(AID-1-T)+SB, LDN, PRM+BDNF (**Figure 2B**).

RT-qPCR: Sh\fP3 has not demonstrated significant differences between control and experimental samples (**Figure 4G**).

ICH: CD133 (↓) and ki67 (↓) (**Figure 4J**), Nestin (↓) and Oct4 (↓) (**Supplementary Figure 3**) decreased expression levels were observed.

G01

RT-qPCR: BMP4 (↓), WNT5 (↓), TNC (↓), L1CAM (↓), bIII-tubulin (↓) expression levels were decreased and CD44 (↑), Notch2 (↑), NeuN (↑) were increased in G01 cell culture (**Figure 4A**).

ICH: decreased expression of CD133 (↓), CD44 (↓), ki67 (↓) and increased expression of NeuN (↑) (**Figure 4D**), decrease in Notch1 (↓), Sox2 (↓), Oct4 (↓) and Nestin (↓) (**Supplementary Figure 3**).

Sus

RT-qPCR: Sus has not shown significant differences in expression of stemness-associated and maturation-associated genes (**Figure 4B**).

ICH: decreased expression of CD133 (↓), CD44 (↓), ki67 (↓) (**Figure 4E**) and Notch1 (↓), Oct4 (↓), Sox2 (↓) (**Supplementary Figure 3**).

40

RT-qPCR: EPHA3 (↑), BMP4 (↑), CD44 (↑), Nestin (↑), Sox2 (↑), Notch2 (↑) and NeuN (↑) expression levels were increased, although there was a decrease in expression level of TNC (↓) (**Figure 4H**).

ICH: decreased expression of CD133 (↓) and bIII-tubulin (↓) (**Figure 4K**), decreased expression of Notch1 (↓) and Nestin (↓), increased expression of Sox2 (↑) (**Supplementary Figure 3**).

Rozh

RT-qPCR: decreased expression of TNC (↓) and increased expression of EPHA3 (↑), BMP4 (↑), CD44 (↑), Sox2 (↑), Notch2 (↑) and NeuN (↑) have been observed (**Figure 4I**).

ICH: ki67 (↓) (**Figure 4L**) and CD133 (↓) (**Supplementary Figure 3**) expression levels were decreased.

*III Grade*

22

MTT: 22 was the most sensitive to such influence among the cell cultures of III Grade. Cell proliferation decreased on 94,5% after exposure to bi-(AID-1-T)+BDNF and on 96,5% after exposure to bi-(AID-1-T)+SB, LDN, PRM+BDNF (**Figure 3A**).

**Figure 5:**
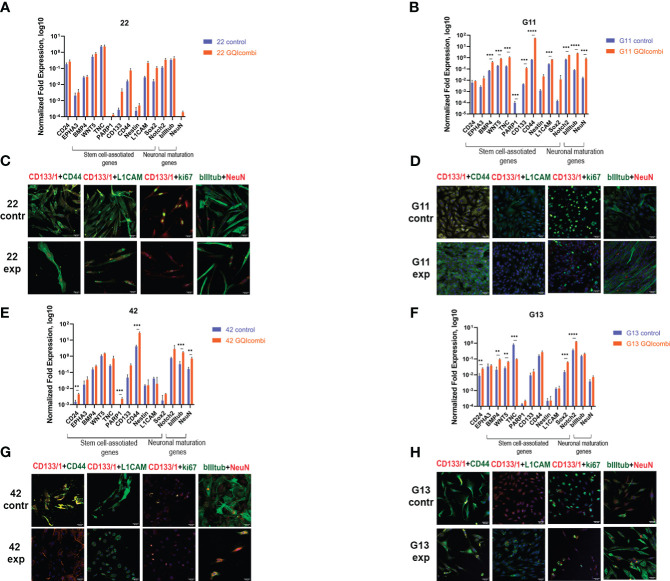
GQIcombi (bi-(AID-1-T) + SB431542, LDN-193189, purmorphamine, and BDNF) influences stemness features of III Grade glioma cell cultures 22, G11, 42 and II Grade glioma cell culture G13. **(A, B, E, F)** The expression of neural stem cells’ genes in 22, G11, 42 and G13 cell cultures in 10 days after the exposure to GQIcombi. Data are represented as mean ± SD; n = 3 for each group. Statistically significant differences between the control and the treatment groups are indicated by asterisks (multiple unpaired t test, *= p<0.05, ** = p<0.01, *** = p<0.001, **** = p<0.0001). **(C, D, G, H)** Micrographs of immunocytochemical staining of 22, g11, 42 and g13 cell cultures with anti-CD133, anti-CD44, anti-L1CAM, anti-ki67, anti-bIIItubulin and anti-NeuN antibodies before (contr) and after (exp) exposure to GQIcombi. Scale bar is 50 μm.

RT-qPCR: there were no substantial changes in gene expression in 22 cell culture (**Figure 5A**).

ICH: ki67 (↓) (**Figure 5C**), CD133(↓) and Sox2 (↓) (**Supplementary Figure 3**) have decreased.

G11

MTT: cell proliferation decreased on 33,3% after exposure to bi-(AID-1-T)+BDNF and on 50% after exposure to bi-(AID-1-T)+SB, LDN, PRM+BDNF (**Figure 3B**).

RT-qPCR: BMP4 (↑), WNT5 (↑), TNC (↑), CD133 (↑), CD44 (↑), L1CAM (↑), Notch2 (↑), NeuN (↑), bIII-tubulin (↑) have increased (**Figure 5B**).

ICH: ki67 (↓) has decreased (**Figure 5D**).

42

MTT: cell proliferation decreased on 68,9% after exposure to bi-(AID-1-T)+BDNF and on 82,6% after exposure to bi-(AID-1-T)+SB, LDN, PRM+BDNF (**Figure 3E**).

RT-qPCR: CD24 (↑), PARP1 (↑), CD44 (↑), NeuN (↑) and bIII-tubulin (↑) were increased in cell culture 42 (**Figure 5E**).

ICH: ki67 (↓), CD44 (↓) and L1CAM (↓) have decreased, NeuN has increased (↑) (**Figure 5G**), CD133(↓) and Oct4 (↓) have decreased (**Supplementary Figure 3**).

*II Grade*

G13

MTT: cell proliferation decreased on 92,3% after exposure to bi-(AID-1-T)+BDNF and on 69,2% after exposure to bi-(AID-1-T)+SB, LDN, PRM+BDNF (**Figure 3F**).

RT-qPCR: there was an increased expression of CD24 (↑), BMP4 (↑), WNT5 (↑), Sox2 (↑), Notch2 (↑) and decreased expression of TNC (↓) (**Figure 5F**).

ICH: decreased expression of ki67 (↓) and increased expression of bIII-tubulin (↑) was observed (**Figure 5H**).

Summarizing the results for IV, III and II Grade gliomas, GQIcombi decreases cell proliferation and stimulated expression of NeuN in most tested cell cultures indicating cell maturation.

### The treatment with GQIcombi leads to apoptotic death of most human glioma tumor cells *in vitro*

**Figure 6:**
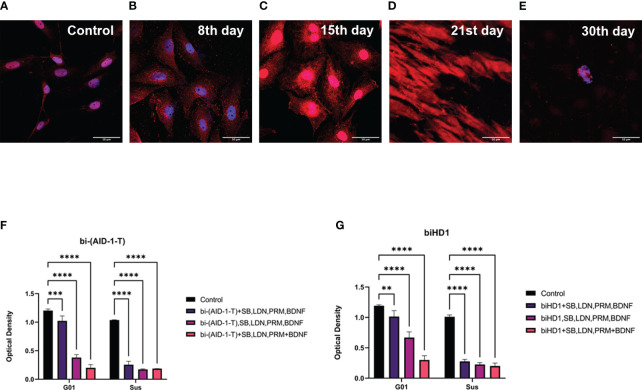
Apoptosis of G01 cells upon GQIcombi (bi-(AID-1-T) + SB431542, LDN-193189, purmorphamine, and BDNF) exposure bi-(AID-1-T) and biHD1 exposure regimes. Staining of glioblastoma cell culture G01 for apoptosis upon exposure to GQIcombi (caspase-3- red, bisbenzimide - blue). Induction of apoptosis in control cells **(A)**, in 8 days **(B)**, 15 days **(C)**, 21 days **(D)** and 30 days **(E)** after the beginning of the experiment. Changing in proliferative rate of G01 and Sus glioma cell cultures by sequential and simultaneous addition of bi-(AID-1-T) **(F)** or biHD1 **(G)** and small molecules. Data are represented as mean ± SD. n=3 for each group. Statistically significant differences between the control and the treatment groups are indicated by asterisks (Two-Way ANOVA, Bonferroni’s multiple comparisons test, ** = p<0.01, *** = p<0.001, **** = p<0.0001). GQ – G-quadruplex, SB - SB431542, LDN - LDN-193189, PRM – purmorphamine.

Since we found a decrease in the proliferative potential of cell cultures of high-grade human gliomas, we analyzed the apoptosis death of tumor cells. The analysis has been performed on G01 glioblastoma cell culture using antibodies against caspase-3, which is an enzymes responcible for apoptosis. The analysis was done after GQIcombi treatment on days 8, 15, 21 and 30 (**Figures 6A–E**). Anti-caspase-3 antibody staining was detected as early as day 8, with a significant increase by day 21 (**Figure 6D**). On the 30th day, only lonely cells were found that did not stain for caspase-3, which were subjected to additional analysis for neuronal differentiation.

### Simultaneous exposure of bi-(AID-1-T) and small molecules is the most effective cell treatment scheme

The expediency of using the sequential addition of bi-(AID-1-T) and small molecules was shown in the experiment with the simultaneous single addition of bi-(AID-1-T) and a cocktail of small molecules to glioma cell cultures and the addition of bi-(AID-1-T) followed by the introduction of a mixture of small molecules. biHD1 was used for comparison as a G-quadruplex with antiproliferative activity (21).

Two combinations of G-quadruplex with antiproliferative properties biHD1 and bi-(AID-1-T) and small molecules LDN-193189, SB431542, Purmorphamine and neurotrophine BDNF were tested in the following regimes:

1. On the 1^st^ day biHD1 or bi-(AID-1-T), on the 3^rd^ day all of the small molecules LDN-193189, SB431542, Purmorphamine and BDNF were added to the culture medium;
2. On the 1^st^ day all the factors were added to the culture medium;
3. On the 1^st^ day biHD1 or bi-(AID-1-T), on the 3^rd^ day LDN-193189, SB431542, on the 5^th^ day Purmorphamine, and on the 7^th^ day BDNF were added to the culture medium.

Upon adding bi-(AID-1-T) (**Figure 6F**) cell culture G01 showed to mostly inhibit its proliferation activity when using the sequential addition of factors (3^rd^ option) - on 76,9%, for the 1^st^ option the decrease was 15%, for the 2^nd^ option - on 43,9%. The addition of biHD1 (**Figure 6G**) led to a decrease in proliferative activity for the 1^st^ variant on 14,5%, for the 2^nd^ – on 68,2%, for the 3^rd^ - on 85,7%.

Cell culture Sus turned out to be more sensitive to the addition of the studied factors. The addition of bi-(AID-1-T) (**Figure 6F**) led to a decrease in proliferation on 75,4% for the 1^st^ variant, for the 2^nd^ - on 83,3%, and for the 3^rd^ - on 82%. With the addition of biHD1 (**Figure 6G**), inhibition of proliferation for the 1^st^ exposure option was 72,6%, for the 2^nd^ – 77,6%, for the 3^rd^ – 77,8%.

Thus, bi-(AID-1-T) added sequentially with the small molecules is the most effective regime that inhibits cell proliferation.

### GQIcombi inhibits growth of 101/8 tumor in rat glioma models

GQIcombi’s impact on glioma cell growth was validated in rat models, enhancing *in vivo* applicability. Rat glioma 101/8 was implanted, followed by bi-(AID-1-T) and doubled small molecule doses. Notably improved results were observed compared to the standard scheme (data not shown).

**Figure 7:**
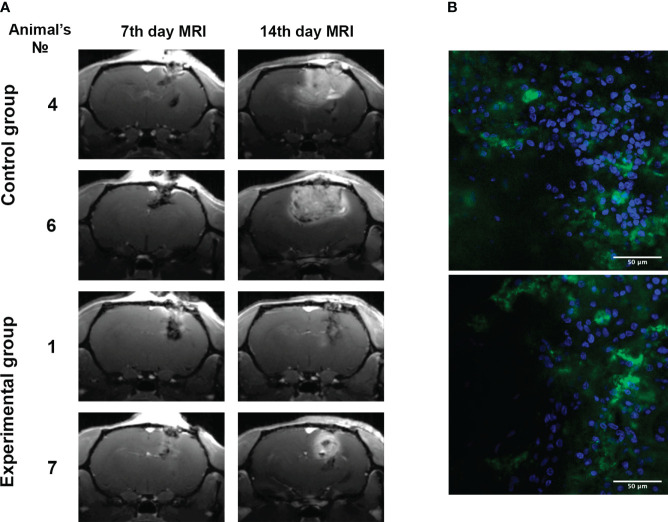
Inhibition of 101/8 tumor growth in rat glioma models upon GQIcombi (bi-(AID-1-T) + SB431542, LDN-193189, purmorphamine, and BDNF) exposure. **(A)** MRI scans of the 7th and 14th days’ (T1-weighted images using Gadovist) animals with implanted glioblastoma 101/8 in the control and experimental groups. **(B)** Accumulation of bi-(AID-1-T)-FAM in a 101/8 tumor in 15 minutes after the injection into the rat tail vein (FAM - green, bisbenzimide - blue).

Remarkably, stable tumor growth inhibition was evident post-therapy (**Table 4**). Control vs. experimental groups had average tumor sizes of 49.6 ± 11.1 mm^3^ and 58.2 ± 29.3 mm^3^ on day 7, and 244.4 ± 60.5 mm^3^ and 45.2 ± 15.3 mm^3^ on day 14, respectively. Control group tumors increased by 392%, while the experimental group’s shrank by 22.3%. **Figure 7A** displays tumor tomograms at maximal area on the section.

**Table 4:** Volumometry of tumor 101/8 according to MRI results.

Group	Animal’s №	Damage volume on day 7, V (mm^3^)	Damage volume on day 14, V (mm^3^)	Changes (times)
Control	3	62,4 ± 2,0	–	–
	4	42,4 ± 2,0	201,6 ± 5,0	4.8 Ý
	6	44,0 ± 2,0	287,2 ± 5,0	6.5 Ý
Experimental	1	40,8 ± 2,0	34,4 ± 2,0	1.2 ß
	7	41,6 ± 2,0	56,0 ± 2,0	1.3 Ý
	8	92,0 ± 4,0	–	**-**

An optimized scheme emerged: bi-(AID-1-T) at 37.5 μM on day 4; bi-(AID-1-T) + SB431542 + LDN-193189 on day 5; SB431542 + LDN-193189 + purmorphamine on day 6; purmorphamine + BDNF on day 7. This strategy suits glioblastoma 101/8 in a rat model, mirroring human glioblastoma characteristics (necrosis, aggressive cell location, vascularity).

Blood-brain barrier penetration was assessed using bi-(AID-1-T)-FAM injected into rat tail veins (**Figure 7B**), revealing significant G-quadruplex accumulation in tumor cells’ cytoplasm and intercellular space. Intravenous tail administration of bi-(AID-1-T)-FAM showcased the aptamer’s blood-brain barrier crossing potential.

## Discussion

Gliomas are composed of highly heterogenic cells, which complicates their therapy. The to-date chemotherapy drugs are aimed at the destruction of actively proliferating cells, thus killing both the cancer cells and the healthy ones, e.g., blood cells or cells of gastrointestinal tract, etc. At this, the toxic effect of chemotherapy is frequently even more life threatening than the tumor itself. Concerning gliomas, the chemical therapeutics known so far have little efficiency against these tumors and their effect does not last long, which leads to tumor relapse. Recurring tumors are usually highly resistant to the drug applied before. These problems make researchers to search for other approaches to cancer therapy, with the complex approaches implying the combination of different drugs to target different types of tumor cells at once being the most obvious ones (2).

However, there is another possible option for malignant tumor therapy named “differentiation therapy”. It relies on the use of specific molecules able to stimulate non-mature cell differentiation to mature ones, rather than on the cytotoxic medications aimed at the destruction of cancer cells. In this way the very proliferation potential of cells is blocked (39, 40). This approach allows to avoid the cytotoxic effect and to target the tumor cells of all stages of differentiation including cancer stem cells (24). The application of GQIcombi for the differential therapy of human glioblastoma proved itself as a promising approach (24).

The first component of the GQIcombi cocktail is a G-quadruplex oligonucleotide bi-(AID-1-T), which possess antiproliferative cytostatic properties for glioma cells (21). The use of the aptamer bi-(AID-1-T) on the first stage of the differential therapy is important, for the actively proliferating glioma cells are unable to differentiate into neural cells in response to small molecules. It is essential to make malignant cells to enter G0 stage of a cells cycle. bi-(AID-1-T) appeared to be an apt agent to cytotoxically stop the cell division cycle. After that we applied to combination of small molecules, namely, SB431542, LDN-193189, purmorphamine, and BDNF (24). SB431542 (SB) is capable to specifically interact with the ATP-binding domains of activin receptor-like kinases ALK5, ALK4, and ALK7, thus it inhibits Smad2/3 activation and blocks TGF-β signal transduction, which affects gene expression (41). It is worth to note that SB induces the differentiation of human ESC, which evidences its potential to affect even completely non-mature cells (42, 43). In our study we used SB simultaneously with LDN-193189 (LDN). The latter is known for the inhibition of BMP4-directed activation of Smad1, Smad5, and Smad8. In fact, LDN inhibit the activation of an alkaline phosphatase. The LDN-driven regulation of BMP cascade is essential for significant stimulation of glioma cell differentiation, as the BMP-induced intracellular pathways are the key ones in the ontogenesis of vertebrates. BMPs regulate gastrulation, pattern formation, and organogenesis; the expression of BMPs defines the fate of multipotent cells (44, 45) Another molecule within GQIcombi therapy is purmorphamine, which affects neuronal differentiation by the control of the Hedgehog pathway (46). In this way purmorphamine targets the group of non-mature cells following the cancer stem cells. The final component of the GQIcombi cocktail is BDNF, which induces Wnt/β-catenin and ERK/MAPK pathways essential for the final neuronal differentiation (47).

We managed to demonstrate the decrease in the proliferation of glioblastoma cells (Grade IV) using several cell cultures obtained from a surgically removed human tissue sample (24). First, we wanted to enlarge the panel of heterogenic cell cultures of glioma by including the tumors of different stages of malignancy. Second, the analysis of the shifts in cell culture transcriptome after GQIcombi was important to pick the genes characteristic for therapy-resistant tumor cells survived after the treatment. We also were interested in the processes within tumor cells after the application of GQIcombi.

GQIcombi was tested on the panel of human glioma cell cultures of Grades II, III, and IV, which differ in their proliferation rates. A significant inhibition of proliferative activity of cells was demonstrated for all of the cell cultures tested (**Figures 2**, **3**). It is worth to mention that we used two methods for the estimation of the proliferative activity, namely, MTT assay and xCelligence S16. The former one relies on the assessing the reduction of MTT (3-(4,5-dimethylthiazol-2-yl)-2,5-diphenyltetrazolium bromide), to formazan, which correlates with the enzymatic activity in living cells’ mitochondria. However, a number of studies report that the oxidative process may also take place in endosomes and lysosomes, thus interfering the correlation with proliferation in cancer cells (48). For this reason, we additionally used xCelligence S16, which is based on the detection of the electric impedance in a well with a given number of cells in a given period of time, which allows to make a curve of cell index over time (49). As a result, the GQIcombi efficiency of application on G13 cell culture (glioma, Grade II) was approved by the xCelligence S16, while the MTT assay showed only a slight decline in the proliferation, which may be due to high enzymatic activity of mitochondria, endosomes, and lysosomes (**Figure 3**). Our study on glioma cell cultures of Grades II-IV clearly demonstrates that the application of the differential therapy based on GQIcombi is a universal and effective approach to target heterogenic glioma cells *in vitro.*

Transcriptome analysis of human glioblastoma cell culture G01 before and after treatment with GQIcombi allowed to design a panel of genes which expression significantly changes after the differential therapy (**Figure 1**). We applied this gene panel for RT-qPCR analysis of GQIcombi effect on all the glioma cell cultures Grades II-IV under study. The RT-qPCR analyses of the human glioma cell cultures demonstrated their high heterogeneity (**Table 5**). It is worth to note that TNC gene expression was reduced in 5 of the 10 cell cultures. TNC gene expression is upregulated during embryogenesis, inflammation-derived pathologies, and oncology (50) In the brain tissues of adults, the expression of TNC is usually low, while neurogenesis-associated regions, such as SVZ (subventricular zone) and SGZ (subgranular zone), where neural stem cells are localized, are characterized by elevated TNC. Overexpression of TNC was detected in malignancies including gliomas.

**Table 5:** Alterations in genes in glioma cell cultures of II, III and IV Grades upon exposure to GQIcombi (bi-(AID-1-T) + SB431542, LDN-193189, purmorphamine, and BDNF).

	CD133	CD44	L1CAM	Sox2	Notch2	Nestin	PARP1	CD24	EPHA3	BMP4	WNT5	TNC	bIII-tub	NeuN
G01	–	↑	↓	–	↑	–	–	–	–	↓	↓	↓	↓	↑
Sus	–	–	–	–	–	–	–	–	–	–	–	–	–	–
Sh\fP3	–	–	–	–	–	–	–	–	–	–	–	–	–	–
40	–	↑	–	↑	↑	↑	–	–	↑	↑	–	↓	–	↑
Bl	–	↓	–	↓	↓	–	↓	–	↓	↓	↓	↓	↓	–
Rozh	↑	↑	–	↑	↑	↑	–	–	–	–	↓	↓	↑	↑
22	–	–	–	–	–	–	–	–	–	–	–	–	–	–
G11	↑	↑	↑	–	↑	–	↓	–	–	↑	↑	↑	↑	↑
42	–	↑	–	–	–	–	↑	↑	–	–	–	–	↑	↑
G13	↑	–	–	↑	↑	–	–	–	–	↑	↑	↓	–	–

↓, decreased; -, not changed; ↑, increased.

(51) TNC is known for endogenously activate TLR4 and promote inflammatory response. In addition to glioma invasion stimulation, TNC was demonstrated to affect tumor-associated microglia. High TNC levels correlate with the poor prognosis for a patient (52) Thus, the decline in the TNC expression after the differential therapy may be a considered highly desirable effect and claims further investigation.

The gene expression analysis based on the estimation of mRNA quantity solely is not reliable. It is necessary to estimate the presence of the expression product, i.e., the protein (**Table 6**). Immunocytochemistry analyses of glioma cell cultures after the treatment with GQIcombi approve the decrease in the expression of ki67 which evidence the decline in the proliferative potential (**Figures 4**, **5**). In addition, upon the action of GQIcombi on human glioma cell culture, the lived cells are stained for βIII-tubulin and NeuN, which evidences their maturation (**Figures 4**, **5**). These results also indicate that after the treatment with GQIcombi the cells differentiate to neural ones, which completely blocks their proliferation. We found that after differential therapy the expression of CD133, Notch1, and Oct4 decreased in 8 cell cultures out of 10, which reflects the effect of the therapy on glioma stem cells.

**Table 6:** Alterations in proteins in glioma cell cultures of II, III and IV Grades upon exposure to GQIcombi (bi-(AID-1-T) + SB431542, LDN-193189, purmorphamine, and BDNF).

	CD133	CD44	L1CAM	ki67	bIII-tub	NeuN	Notch1	Sox2	Oct4	Nestin
G01	↓	↓	–	↓	–	↑	↓	↓	↓	↓
Sus	–	↓	–	↓	↑	–	↓	↓	↓	–
Sh\fP3	↓	–	–	↓	–	–	–	–	↓	↓
40	↓	–	–	–	↓	–	↓	↑	–	↓
Bl	–	–	–	↓	↑	↓	↓	–	↓	–
Rozh	↓	–	–	↓	↑	↓	–	–	–	–
22	↓	–	–	↓	–	–	–	↓	–	–
G11	–	–	–	↓	–	–	↓	↑	↓	–
42	↓	↓	↓	–	–	↑	–	–	–	–
G13	↑	–	–	↓	↑	–	–	–	–	–

↓, decreased; -, not changed; ↑, increased.

At this, it is obvious that not all of the glioma tumor cells are able to differentiate into neural cells in response to GQIcombi. The decline in the proliferation rates of tumor cells after the application of GQIcombi observed on xCelligence let us assume that some of glioma cells undergo apoptosis. Our results (**Figure 6**) confirm this. As early as by day 8 after the addition of the factors into the culture medium human glioma cells are stained for caspase-3 reaching the maximum apoptosis rate by day 21 after the treatment. In the cells survived the GQIcombi the neural differentiation pathway is induced, which is confirmed by bIII-tubulin and NeuN staining.

Besides, we demonstrated that the successive addition of the G-quadruplex aptamer and the small molecules is of principal significance, as it consecutively induces different pathways of neural differentiation in non-mature progenitor cells (**Figure 6**).

To estimate the effectiveness of *in vivo* application of GQIcombi we used intracranially implanted 101/8 glioma in rat as a model. First, we had to prove that bi-(AID-1-T) targets brain cells and is accumulated in the tumor. This was clearly shown in the experiment with the systemic injections of GQIcombi into the tail vein of rats demonstrating that the G-quadruplex penetrates the blood-brain barrier (**Figure 7**), which makes the application of injections possible in further clinical practice.

In the animal studies we also demonstrated that the GQIcombi treatment of glioma cells implanted into the brain of rats significantly reduced the tumor growth compared to control animals (**Figure 7**). Thus, the combination under study is applicable not only for *in vitro* models, but also for *in vivo* studies.

In sum of the abovesaid, the GQIcombi cocktail has a universal antiproliferative effect on glioma tumor cells. The differential therapy may become a cardinal and successful approach to the human glioma issue. Moreover, we believe that the approach is applicable to a wide range of tumors. For instance, following our publication in (24) which was preprinted on the Research Square platform (53), Xu Zhang et al. (54), reported the application of the differential therapy with the small molecules for liver cancer. It is worth to note that they also used SB431542 and LDN193189 to induce cell maturation. At the same time, a number of problems in the work may be explained by the lack of a cytostatic factor to stop cell proliferation on the first stage. Such an unanimity evidences the significant potential of the differential therapy as an approach to cancer treatment, which, in addition to its effectiveness, will be less traumatic for a patient.

## Data availability statement

The datasets presented in this study can be found in online repositories. The names of the repository/repositories and accession number(s) can be found below: https://www.ncbi.nlm.nih.gov/geo/query/acc.cgi?acc=GSE250617.

## Ethics statement

The studies involving humans were approved by The Local Ethics Committee of Burdenko Neurosurgery Center. The studies were conducted in accordance with the local legislation and institutional requirements. The participants provided their written informed consent to participate in this study. The animal study was approved by the Ethics Committee of the Institute of Higher Nervous Activity and Neurophysiology of Russian Academy of Science. The study was conducted in accordance with the local legislation and institutional requirements.

## Author contributions

VK: Conceptualization, Formal analysis, Investigation, Methodology, Visualization, Writing – original draft, Writing – review & editing. ARe: Investigation, Writing – review & editing. LF: Investigation, Writing – review & editing. AA: Investigation, Writing – review & editing. ARy: Investigation, Writing – review & editing. IP: Resources, Writing – review & editing. DU: Resources, Writing – review & editing. AK: Supervision, Writing – review & editing. GP: Conceptualization, Funding acquisition, Methodology, Project administration, Resources, Supervision, Validation, Visualization, Writing – original draft, Writing – review & editing.
