# Supplementary material for: GQIcombi application to subdue glioma via differentiation therapy
Source: Front Oncol. 2024 Jun 26;14:1322795. doi: 10.3389/fonc.2024.1322795 (PMC11233813; doi:10.3389/fonc.2024.1322795)
Supplement: Supplementary file 1 [file DataSheet_1.pdf]

frontiers

Supplementary Material

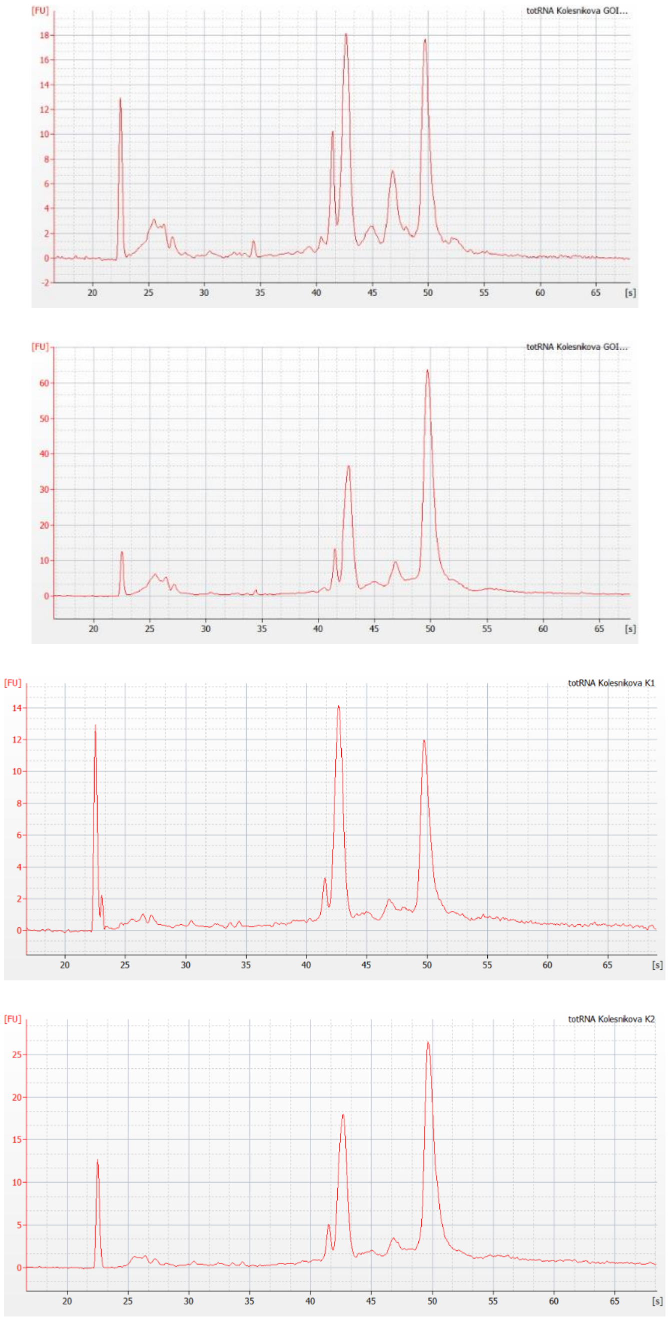

Supplementary Figure 1. Fragment length distribution in isolated RNA.

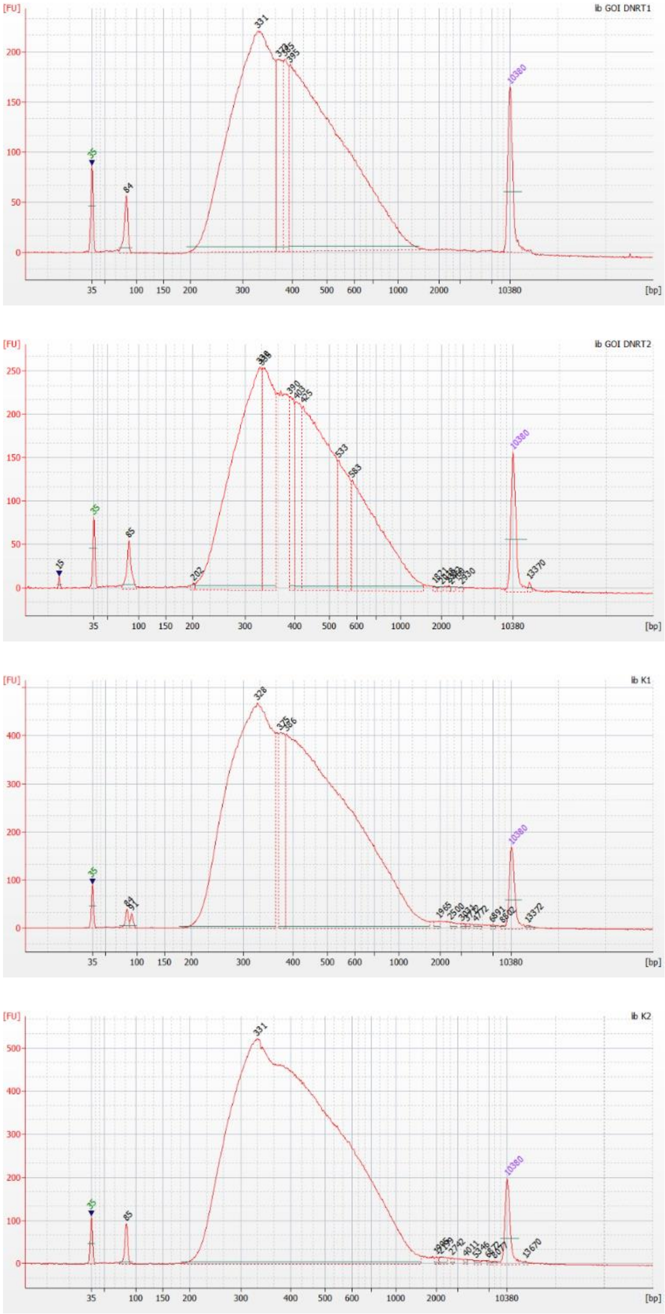

Supplementary Figure 2. Distribution of fragment lengths of the resulting libraries.

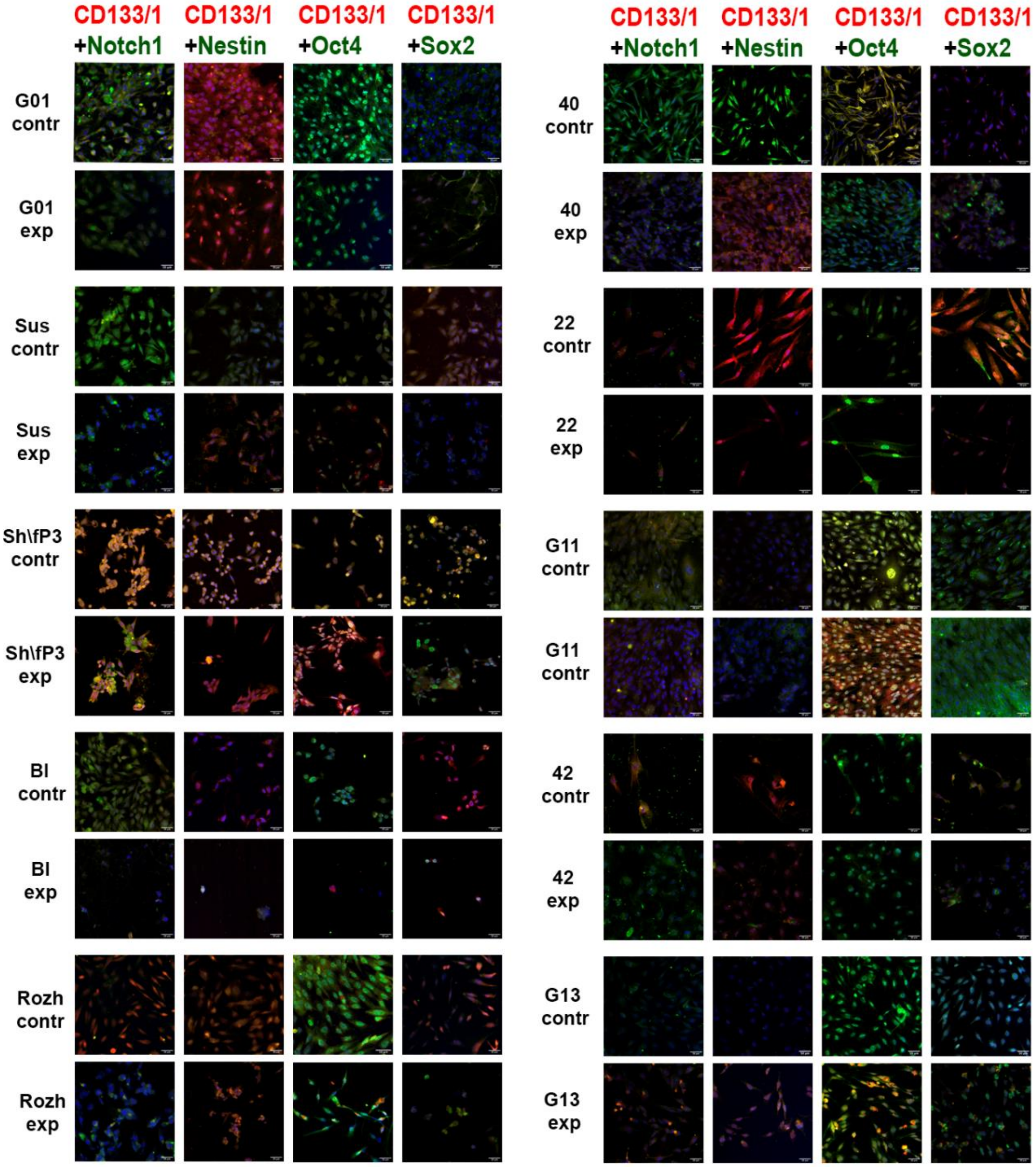

Supplementary Figure 3. Immunocytochemical staining of IV, III and II glioma cell cultures.

Micrographs of immunocytochemical staining of II-IV grades' glioma cell cultures with anti-CD133, anti-Notch1, anti-Nestin, anti-Oct4 and anti-Sox2 antibodies before (contr) and after (exp) exposure to GQIcombi (bisbenzimide - blue). Scale bar is 50 µm.

Sample name	Libraries' concentrations, ng\µl
K1	11,8
K2	11,8
GOI_DNRT1	4,77
GOI_DNRT2	6,5

Supplementary Table 1. Libraries' concentrations.

Sample name	Number of reads
GOI_DNRT1	11233275
GOI_DNRT2	10556041
K1	11778375
K2	10506385

Supplementary Table 2. The number of reads for each sample.
